# Direct Interaction between EgFABP1, a Fatty Acid Binding Protein from *Echinococcus granulosus*, and Phospholipid Membranes

**DOI:** 10.1371/journal.pntd.0001893

**Published:** 2012-11-15

**Authors:** Jorge L. Porfido, Gabriela Alvite, Valeria Silva, Malcolm W. Kennedy, Adriana Esteves, Betina Corsico

**Affiliations:** 1 Instituto de Investigaciones Bioquímicas de La Plata, Facultad de Ciencias Médicas, Universidad Nacional de La Plata, La Plata, Buenos Aires, Argentina; 2 Consejo Nacional de Investigaciones Científicas y Técnicas (CONICET), Ciudad Autónoma de Buenos Aires, Argentina; 3 Sección Bioquímica, Facultad de Ciencias, Universidad de la República, Montevideo, Uruguay; 4 Institute of Molecular, Cell and Systems Biology, and Institute of Biodiversity, Animal Health and Comparative Medicine, College of Medical, Veterinary and Life Sciences, University of Glasgow, Glasgow, United Kingdom; Institut Pasteur de Montevideo, Uruguay

## Abstract

**Background:**

Growth and maintenance of hydatid cysts produced by *Echinococcus granulosus* have a high requirement for host lipids for biosynthetic processes, membrane building and possibly cellular and developmental signalling. This requires a high degree of lipid trafficking facilitated by lipid transporter proteins. Members of the fatty acid binding protein (FABP) family have been identified in *Echinococcus granulosus*, one of which, EgFABP1 is expressed at the tegumental level in the protoscoleces, but it has also been described in both hydatid cyst fluid and secretions of protoscoleces. In spite of a considerable amount of structural and biophysical information on the FABPs in general, their specific functions remain mysterious.

**Methodology/Principal Findings:**

We have investigated the way in which EgFABP1 may interact with membranes using a variety of fluorescence-based techniques and artificial small unilamellar vesicles. We first found that bacterial recombinant EgFABP1 is loaded with fatty acids from the synthesising bacteria, and that fatty acid binding increases its resistance to proteinases, possibly due to subtle conformational changes induced on EgFABP1. By manipulating the composition of lipid vesicles and the ionic environment, we found that EgFABP1 interacts with membranes in a direct contact, collisional, manner to exchange ligand, involving both ionic and hydrophobic interactions. Moreover, we observed that the protein can compete with cytochrome c for association with the surface of small unilamellar vesicles (SUVs).

**Conclusions/Significance:**

This work constitutes a first approach to the understanding of protein-membrane interactions of EgFABP1. The results suggest that this protein may be actively involved in the exchange and transport of fatty acids between different membranes and cellular compartments within the parasite.

## Author Summary

*Echinococcus granulosus* is the causative agent of hydatidosis, a zoonotic infection that affects humans and livestock, representing a public health and economic burden in many countries. Since the parasites are unable to synthesise most of their lipids *de novo*, they must acquire them from the host and then deliver them by carrier proteins to specific destinations. *E. granulosus* produces in abundance proteins of the fatty acid binding protein (FABP) family, one of which, EgFABP1 has been characterised at the structural and ligand binding levels, but it has not been studied in terms of the mechanism of its interaction with membranes. We have investigated the lipid transport properties and protein-membrane interaction characteristics of EgFABP1 by applying biophysical techniques. We found that EgFABP1 interacts with membranes by a mechanism which involves direct contact with them to exchange their cargo. Given that the protein has been found in the secretions of the parasite, the implications of its direct interactions with host membranes should be considered.

## Introduction

Hydatidosis is a highly pathogenic infection with an almost global incidence caused by the larval stage (metacestode) of the cestode *Echinococcus granulosus*. In endemic areas it has serious health effects on humans, livestock and wildlife, representing a major public health and economic burden in many countries [1]–[3]. *Echinococcus* species, as do other tapeworms of mammals, require two hosts to complete their life cycle. The *E. granulosus* eggs containing the infective oncosphere are shed in the faeces of wild and domestic carnivores that are the definitive hosts harbouring the dwarf adult tapeworms. Once a suitable intermediate host ingests the eggs, they hatch and the oncosphere is released, escaping from the intestine to establish hydatid cysts in liver and lungs. The cyst produces thousand of protoscoleces, each of which can progress to the adult form when ingested by the definitive host [4], but it is the hydatid cysts in intermediate hosts that cause significant pathology and death. Hydatid disease in humans is highly pathogenic and is particularly difficult to treat successfully, especially so when cysts develop and proliferate in the lungs.

Fatty acid binding proteins (FABPs) are small proteins (14–15 kDa) that bind non-covalently to hydrophobic ligands, mainly fatty acids (FA) and retinoids. FABPs are confined to the interior of the synthesising cells, the only known exceptions to this being in nematodes [5], [6]. Several tissue-specific FABP types have been identified in vertebrates, each named after the tissue in which they are predominantly expressed, and have also been given numeric designations [7]. In mammals they are implicated in intracellular uptake, storage and transport of FAs in lipid metabolism and membrane building, as well as protection from the membrane-disruptive effects of free long chain FAs [8]. In addition, the non-FA-binding retinoid-binding isoforms contribute to regulation of gene expression [9]. However, the precise function of each FABP type remains poorly understood, but sub-specialization of functions is suggested by the tissue-specific and temporal expression, in addition to ligand preferences [10]. Despite very similar tertiary structures, FABPs have been found to interact with membranes in different ways that might reflect how they acquire and deliver their cargoes. The fluorescence-based biophysical approaches used for this have shown that most FABPs from mammals (adipocyte FABP, intestinal FABP, heart FABP, keratinocyte FABP, myelin FABP, etc.) and one from Schistosomes (Sj-FABPc) exhibit a collisional mechanism of ligand exchange, meaning that they interact by direct contact with a membrane in ligand transfer. In contrast, only liver FABP and cellular retinol binding protein II from mammals transfer ligands in a diffusional mechanism, meaning that transfer occurs without requiring direct contact between protein and membrane but through release of ligand into the aqueous phase followed by its intercalation into the membrane. Proteins like liver FABP may therefore be more involved in lipid storage and regulation in the cytoplasm rather than in direct transport of FAs [8], [11], [12].

FABPs of parasitic platyhelminths are interesting because these parasites are unable to synthesise most of their own lipids *de novo*, in particular long-chain FAs and cholesterol [13], [14]. Such lipids must therefore be acquired from the host, and then delivered by carrier proteins to specific destinations within the parasite. Whether they are involved extracellularly in lipid acquisition from, or delivery to, host cells, remains to be seen. It is noteworthy that EgFABP1 has been found in hydatid cyst fluid and in protoscolex secretions [15], [16]. A final reason for interest in FABPs is their potential role in drug delivery and the fact that they have been assayed as vaccine candidates [17]–[23].

EgFABP1 is considered to be a member of the heart FABP subfamily [24], [25], whose members are believed to be involved in lipid oxidation processes [8]. The ligand-binding properties of EgFABP1 have been investigated by the displacement of cis-parinaric acid by a set of hydrophobic ligands [26], and its crystal structure reveals the 10-stranded β-barrel fold typical of the family of intracellular lipid-binding proteins [27].

The objective of this study was to investigate the lipid transport properties and protein-membrane interaction characteristics of EgFABP1. We characterise the biophysical properties of the protein in a number of ways, and show that the protein exchanges FAs through a collisional, direct contact, mechanism with acceptor membranes, indicating that it may indeed be involved in FA dynamics within the parasite, but that it may also engage in direct, non-specific interactions with host cell membranes.

## Materials and Methods

### Production of recombinant EgFABP1

The cDNA encoding EgFABP1 (UniProtKB/Swiss-Prot Q02970) was subcloned into pET11b. The expression of the protein was carried out in *E. coli BL21(DE3)* by induction with 0.4 mM isopropyl-beta-D-thiogalactoside for 3 hours at 37°C in Luria Bertani medium in presence of 100 µg/mL of ampicillin. Cells were lysed by sonication and the lysate clarified by ultracentrifugation (25 min, 61700× g, 4°C). Following clarification, the supernatant was subjected to salting out incubating the protein for 2 hours at 4°C with 0.5 volume of a saturated ammonium sulphate solution. After centrifugation, the obtained protein solution was applied into a size exclusion chromatographic column (Sephadex G-50, Pharmacia Biotech Inc.). The fractions containing EgFABP1 were subsequently subjected to ionic exchange chromatography employing a MonoQ column (Pharmacia Biotech Inc.) in order to remove nucleic acids contamination. Delipidation was carried out using a Lipidex 1000 column (Sigma) at 37°C in a high ionic strength buffer (10 mM phosphate (K_2_HPO_4_ 6 mM+KH_2_PO_4_ 4 mM), 1 M KCl).

### Analysis of EgFABP1-bound fatty acids

As an approach for studying binding preferences of EgFABP1, the lipid moiety of recombinant non-delipidated EgFABP1 was extracted according to Bligh & Dyer's method [28] and analysed on a TLC plate using a mobile phase for resolving neutral lipids (hexane∶diethyl-ether∶acetic acid at 80∶20∶1, v∶v∶v). The FA composition of EgFABP1 lipid fraction was analysed by GC of their methyl esters derivatives methylated with BF_3_-Methanol according to the method described by Morrison & Smith [29], employing an HP 6890 device Hewlett Packard) as described previously by Maté et al. [30].

### Limited proteolysis

In order to analyse possible conformational changes between apo- and holo- forms, EgFABP1 was subjected to limited proteolysis experiments. The protocol was a modification of that described by Arighi et al. [31]. Briefly, clostripain (ArgC, Sigma) was activated by preincubation in 10 mM phosphate (K_2_HPO_4_ 6 mM+KH_2_PO_4_ 4 mM), 150 mM KCl, pH 7.4 and 1 mM DTT for 2 hours. Prior to digestion, delipidated EgFABP1 (0,5 mg/ml) was incubated for 30 min with either myristic acid, palmitic acid, stearic acid or oleic acid in ethanol (4∶1 mol∶mol ligand∶protein) to obtain holo-forms. As a control of the FA solvent used, an equal volume of ethanol was added to the apo-form. Additional 15 min incubation with 1 mM DTT was carried out previous to the addition of the protease. At fixed intervals, samples were collected and frozen for subsequent analysis by SDS-PAGE. SDS-PAGE was carried out according to Schägger and von Jagow [32] in 16.5% acrylamide Tris-Tricine. After Coomassie Blue staining digital images were collected employing an ImageQuant 350 device (GE Healthcare).

### *In vitro* binding properties of EgFABP1

Fatty acid binding to EgFABP1 was assessed employing a fluorescent titration assay [33]. Briefly, 0,5 µM anthroyloxy-fatty acid (AOFA, Molecular Probes) was incubated at 25°C for 3 min in 40 mM Tris, 100 mM NaCl, pH 7.4 buffer (TBS) with increasing concentrations of EgFABP1. The AOFAs employed for binding assays were 12-(9-anthroyloxy)stearic acid (12AS) and 16-(9-anthroyloxy)palmitic acid (16AP). Fluorescence emission at 440 nm was registered after excitation at 383 nm in a Fluorolog-3 Spectrofluorometer (Horiba-Jobin Yvon). An exact equilibrium n-sites binding model was fitted to fluorescence data (using Microcal ORIGIN software) as previously described [34].

### Vesicle preparation

For AOFA transfer experiments, small unilamellar vesicles (SUVs) were prepared by sonication and ultracentrifugation as described previously [35]. The standard vesicles were prepared to contain 90 mol % of egg phosphatidylcholine (EPC) and 10 mol % of *N*-(7-nitro-2,1,3-benzoxadiazol-4-yl)-phosphatidylcholine (NBD-PC), which served as the fluorescent quencher. To increase the negative charge density of the acceptor vesicles, either 25 mol % of phosphatidylserine (PS) or cardiolipin (CL) was incorporated into the SUVs in place of an equimolar amount of EPC. Vesicles were prepared in TBS except for SUVs containing CL which were prepared in TBS with 1 mM EDTA. SUVs containing 64 mol % EPC, 10 mol % egg phosphatidylethanolamine (EPE), 25 mol % CL and 1 mol % dansyl-phosphatidylethanolamine (DPE) were prepared in 20 mM Tris, 0.1 mM EDTA, pH 7.4 for protein-membrane interaction assays.

Large unilamellar vesicles (LUVs) of EPC were prepared (1 mM in phospholipids) by extrusion through polycarbonate membranes of 100 nm pore diameter (Avestin Inc., Ottawa, Canada) as described previously [35]. All lipids were purchased from Avanti Polar Lipids.

### Relative partition coefficient (K_P_) determination

Ligand partition between the protein and NBD-containing SUVs was determined by measuring AOFA fluorescence at different protein∶SUVs ratios obtained by adding SUV to a solution containing 10 µM EgFABP1 and 1 µM 12AS in buffer TBS at 25°C [36]. The relative partition coefficient (K_P_) was defined as:
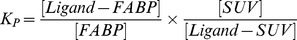
(1)Where [Ligand-SUV] and [Ligand-FABP] are the concentration of AOFA bound to membrane and EgFABP1, respectively, and [FABP] and [SUV] are the concentration of protein and vesicles. The decrease in AOFA fluorescence as a function of SUV is related to K_P_ by
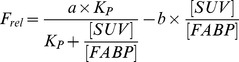
(2)Where F_rel_, [SUV], [FABP], K_p_, a and b are the relative fluorescence, the molar concentration of SUV, the molar concentration of EgFABP1, the partition constant and fitting parameters, respectively [37]. The partition coefficient was used to establish AOFA transfer assay conditions that ensure essentially unidirectional transfer, as detailed below.

### Transfer of AOFA from EgFABP1 to SUV

A Förster Resonance Energy Transfer assay was used to monitor the transfer of 12AS from EgFABP1 to acceptor model membranes as described in detail elsewhere [11], [33], [38]. Briefly, EgFABP1 with bound 12AS was mixed at 25°C with SUVs, prepared as above, using a stopped-flow RX2000 module (Applied Photophysics Ltd.) attached to the spectrofluorometer. The NBD moiety is an energy transfer acceptor of the anthroyloxy group donor; therefore, the fluorescence of the AOFA is quenched when the ligand is bound to SUVs that contain NBD-PC. Upon mixing, transfer of AOFA from protein to membrane is directly monitored by the time-dependent decrease in anthroyloxy group fluorescence. Different SUVs and buffer compositions were employed in order to analyse the ligand transfer mechanism. Transfer assay conditions were 15∶1 mol∶mol EgFABP1∶AOFA ratio. SUVs were added ranging from 1∶10 mol∶mol to 1∶40 mol∶mol EgFABP1∶SUVs. Controls to ensure that photobleaching was eliminated were performed prior to each experiment, as previously described [38]. Data were analysed employing SigmaPlot and all curves were well described by an exponential decay function. For each experimental condition within a single experiment, at least five replicates were done.

### EgFABP1 interaction with membranes

To analyse the putative association of EgFABP1 with vesicles, an assay that exploits the well known membrane-interactive properties of cytochrome c was employed. The binding of cytochrome c to acidic membranes can be monitored by using a resonance energy transfer assay [39] in which the dansyl fluorescence of DPE-labelled SUV is quenched upon binding of cytochrome c, which contains the heme moiety quencher. Competition of EgFABP1 with cytochrome c for binding to SUVs was determined by the relief of cytochrome c-related quenching of the dansyl fluorescence. In a final volume of 200 µl, 0–48 µM EgFABP1 was added to 15 µM SUV in 20 mM Tris.HCl/0.1 mM EDTA, pH 7.4. After a 2 min equilibration, fluorescence emission at 520 nm was measured (λ_ex_ = 335 nm). Cytochrome c (Sigma) was then added (1 µM final concentration), and the mixture equilibrated an additional 2 min period before monitoring again fluorescence emission at 520 nm. In the absence of bound FABP, the dose-dependent quenching of dansyl fluorescence is observed. An inhibition of cytochrome c-dependent quenching is interpreted as evidence for EgFABP1 interaction with SUVs, i.e., EgFABP1 prevention of subsequent cytochrome c interaction with the bilayer.

## Results

### Lipid binding by EgFABP1 in a cellular environment

**Figure 1:**
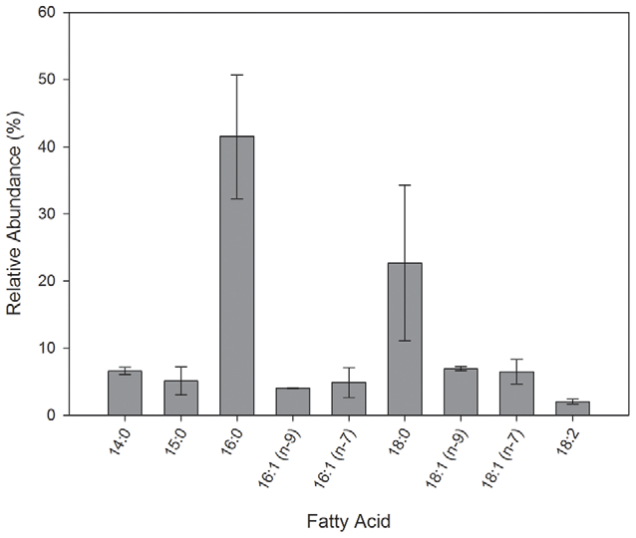
Analysis of FABP-bound FAs. GC analysis of EgFABP1-bound FAs from *E. coli* was performed as indicated in Materials and Methods. Fatty acids detected were: myristic (14:0), pentadecanoic (15:0), palmitic (16:0), 7-hexadecenoic (16:1 n-9), palmitoleic (16:1 n-7), stearic (18:0), oleic (18:1 n-9), vaccenic (18:1 n-7), and linoleic acid (18:2). Average of two different experiments ± SD are shown.

This assay was performed in order to determine which lipid classes bind to EgFABP1 in a cellular environment. Despite *E. coli*'s cytoplasm not being the natural environment of EgFABP1, this approach could contribute to the assignment of the protein's natural ligands as it analyses the preference of EgFABP1 for different hydrophobic compounds present in the bacterial cytoplasm. TLC analysis showed that only FAs were bound to the recombinant protein (data not shown). Among them, palmitic acid (16:0) and stearic acid (18:0) are important ligands, although myristic (14:0), pentadecanoic (15:0), palmitoleic (16:1 n-7), 7-hexadecenoic (16:1 n-9), oleic (18:1 n-9), vaccenic (18:1 n-7), and linoleic acid (18:2) were also detected (Figure 1). The latter may come from culture media, as *E. coli* is not able to synthesise polyunsaturated FAs, at least during log-phase growth [40], [41]. The distribution of FAs bound to FABP may be related to the relative abundance of each of them in *E. coli*, and it correlates well with the reported FA composition of *E. coli* grown in equivalent conditions [41]. As in previous in vitro displacement of fluorescent ligand studies where palmitic and stearic acids are among those that produce moderate displacement percentages (>50%) [26], this experiment shows that EgFABP1 is able to bind many FAs of different chain length and degree of insaturation. In addition, in agreement with these results, the crystal structure of recombinant EgFABP1 revealed an electronic density inside the cavity, which was interpreted as being palmitic acid [27]. We therefore proceeded to investigate protein:membrane transfer using fluorophore-tagged fatty acid analogues.

### Ligand-induced conformational changes

**Figure 2:**
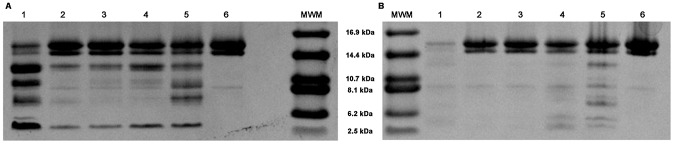
Proteolysis of free EgFABP1 and EgFABP1 bound to ligands. Separation by SDS-PAGE of proteolytic fragments of apo-EgFABP1 (lane 1) and samples of this protein preincubated for 30 min with myristic acid (lane 2), palmitic acid (lane 3), stearic acid (lane 4) or oleic acid (lane 5) to obtain holo-forms, as indicated in Materials and Methods. Lane 6 corresponds to undigested EgFABP1. Samples were taken after 5 min (A) or 16 hours of proteolysis (B). A peptide molecular weight marker is shown in lanes labelled as MWM. This is a representative experiment from two independent experiments.

Partial proteolysis can provide information related to conformational changes in proteins since this technique may reveal the differential exposure of proteolytic sites in *apo* and *holo* forms. We analysed the peptide pattern obtained by digestion of EgFABP1 in its apo- or different holo-forms with Clostripain (ArgC). The FAs selected, following to the analysis of ligands bound to recombinant EgFABP1 (Figure 1), were myristic, palmitic, stearic and oleic acids. The enzyme hydrolyses the polypeptide chain at the C-terminal end of arginine residues. Qualitative differences were evident between apo-EgFABP1 and the different complexes (Figure 2). Results show that binding of FAs gives EgFABP1 significant relative protection against cleavage. After 5 minutes of proteolysis the apo-protein shows several bands corresponding to proteolytic fragments, while the holo-forms show mainly the band corresponding to full-length EgFABP1 and less intense bands corresponding to proteolytic fragments (Figure 2A). This suggests that ligand-binding results in a different exposure of proteolytic sites. It is interesting to note that after 16 hours of proteolysis the holo-proteins do not seem to be further proteolysed while the apo-protein is almost completely degraded (Figure 2B). Previous results obtained for other members of the family of FABPs have suggested that binding of ligands involves conformational changes, especially on the portal region of FABPs [10], [31], [42]. Furthermore, *in silico* simulations show that, upon ligand binding, subtle conformational changes can be detected inside the cavity, in the surface and in the portal region of EgFABP1 (Esteves, unpublished data). These changes could make cleavage sites less accessible to the protease.

As an additional approach to investigate conformational changes between apo- and holo-protein, we analysed the circular dichroism (CD) spectra of EgFABP1 in the far (200–250 nm) and near (250–320 nm) UV regions. Two different ligands were employed for the generation of holo-EgFABP1: palmitic and oleic acid. Results indicated that the far-UV spectra of apo- and the two holo-forms did not show appreciable differences as can be seen in Figure S1. These data could be interpreted to show that no significant changes in overall secondary structure content are caused by ligand binding. On the other hand, the near-UV CD spectra (Figure S1) showed differences upon ligand binding, especially with oleic acid, indicating a likely alteration in the environment of aromatic residues resulting from proximity to ligand and/or a change in the conformation of the protein. So, ligand binding to EgFABP1 could elicit a change in the tertiary structure of the protein that could be correlated to the relative resistance of the holo form to proteolytic attack observed in the previous experiment.

### *In vitro* binding of fluorescent FAs by EgFABP1

**Figure 3:**
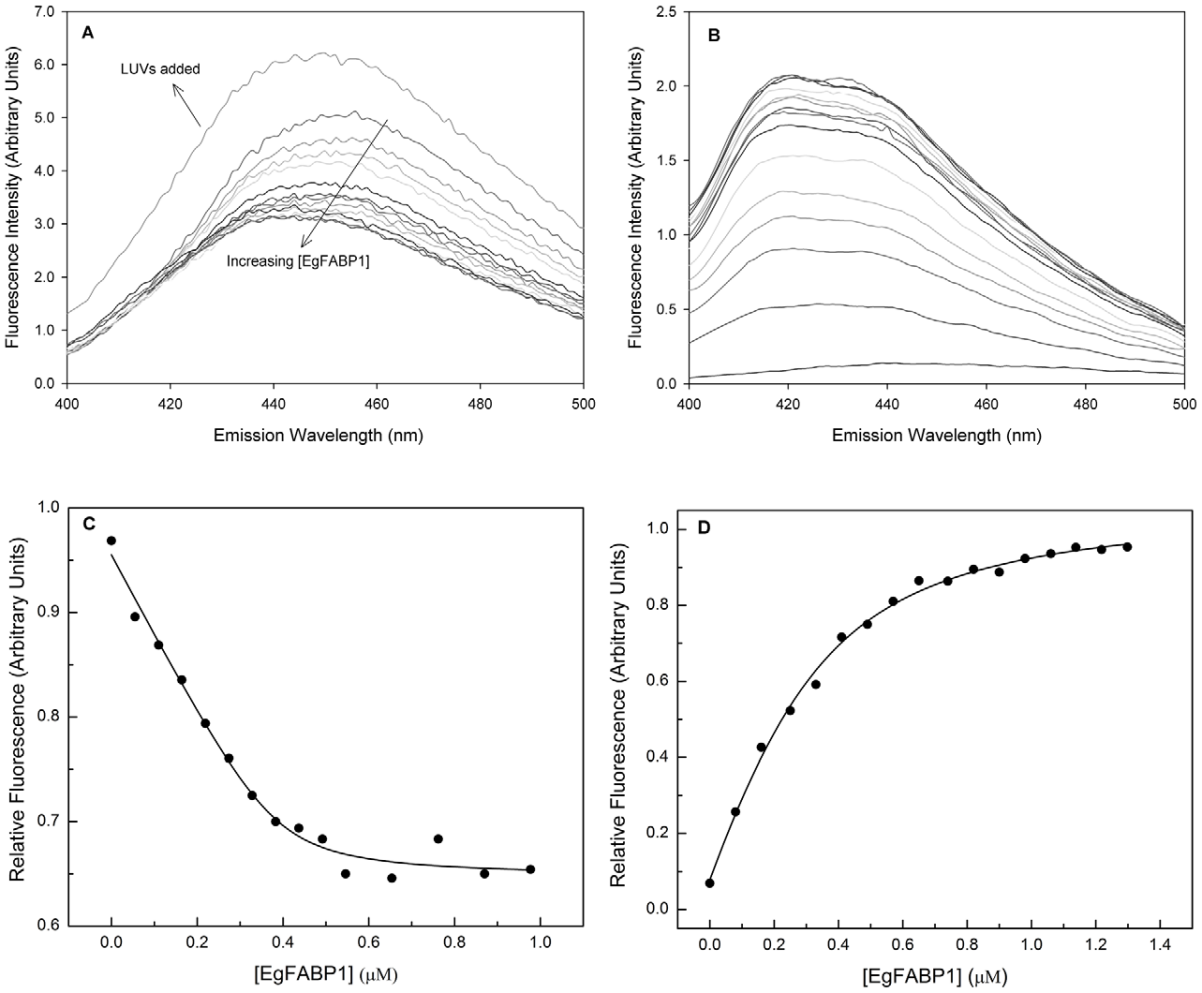
Fluorimetric titration of fluorescent fatty acid analogues with EgFABP1. (A) Emission spectra of 16AP bound to EgFABP1 is shown. Changes in relative 16AP fluorescence were monitored from 400 to 500 nm after excitation at 383 nm upon incremental 0,05 µM additions of EgFABP1 to a cuvette initially containing 2 mL of 0,5 µM 16AP in TBS buffer. 16AP emission spectra show a remarkable blue shift that accompanies fluorescence decrease upon binding to EgFABP1. Fluorescence is recovered after the addition of 10 µM EPC large unilamellar vesicles (LUVs). (B) Emission spectra of 12AS bound to EgFABP1 are shown. Changes in relative 12AS fluorescence were monitored from 400 to 500 nm after excitation at 383 nm upon incremental 0.05 µM additions of EgFABP1 to a cuvette initially containing 2 mL of 0.5 µM 12AS in TBS buffer. 12AS spectra show a remarkable blue shift that accompanies fluorescence increase. (C) Changes in relative 16AP fluorescence were recorded at 440 nm in order to build the binding isotherm. The data are consistent with one binding site per monomer unit of protein and a K_d_ value of 0.013±0.006 µM. The solid line is the theoretical binding curve for complex formation. One representative experiment of three is shown. (D) Changes in relative 12AS fluorescence were recorded at 440 nm to build the binding isotherm of the complex EgFABP1-12AS. The data are consistent with one binding site per monomer unit of protein and a K_d_ value of 0.12±0.02 µM. The solid line is the theoretical binding curve for complex formation. One representative experiment of four is shown.

In preparation for experiments on the interaction of EgFABP1 with phospholipid vesicles, binding experiments were performed using fluorescent analogues of stearic and palmitic acids, 12AS and 16AP, respectively. Anthroyloxy probes are useful indicators of binding site characteristics because their spectral properties are environment-sensitive. These probes usually have very low fluorescence intensity in buffer, which becomes dramatically enhanced upon interaction with a FABP [43]. 12AS showed a large increase in fluorescence emission accompanied by a substantial blue shift upon binding to EgFABP1. On the other hand, 16AP's fluorescence was surprisingly decreased when bound to EgFABP1, but also accompanied by a distinct blue shift in emission (Figure 3). This blue-shift indicates that the fluorophore had entered an apolar environment, almost certainly the hydrophobic binding pocket rather than a superficial, non-specific site of the protein. Following addition of artificial 100 mol % phosphatidylcholine LUVs to the 16AP:EgFABP1 complex, the intensity of fluorescence emission increased, indicating that the quenching of 16AP's fluorescence emission was reversed upon transfer to the different, lipidic, environment of the vesicles. In both cases (12AS and 16AP) the titration described curves that reached saturation, in accordance to a ligand binding phenomenon consistent with 1∶1 binding, with a K_d_ of 0.12±0.02 µM for 12AS, and 0.013±0.006 µM for 16AP. 12AS was chosen as a ligand for the following analysis of transfer kinetics due to its fluorescence emission characteristics when bound to protein being more typical of that observed in other studies on protein to membrane transfer [37], [44], [45]. However, the quenching effect observed with 16AP will be very useful to analyse FA transfer between EgFABP1 and other proteins that show a typical increase of AOFA fluorescence upon binding. Regarding this, another lipid binding protein from *E. granulosus* which is very abundant in the hydatid fluid, Antigen B, has been investigated in its binding properties, showing that it binds 16AP with a 30-fold fluorescence enhancement of the probe [46].

### Relative partition coefficient (K_P_) determination

The apparent partition coefficient that describes the relative distribution of 12AS between EgFABP1 and EPC-SUVs was determined by adding SUVs containing NBD-PC to a solution of 12AS:EgFABP1 complex. As a result of this experiment, a K_P_ value of 0.48±0.23 was obtained employing Eq. 2 (see Materials and Methods), which indicates that there is preference of the AOFA for the phospholipid membranes.

### Effect of vesicle concentration on AOFA transfer from EgFABP1 to membranes

**Figure 4:**
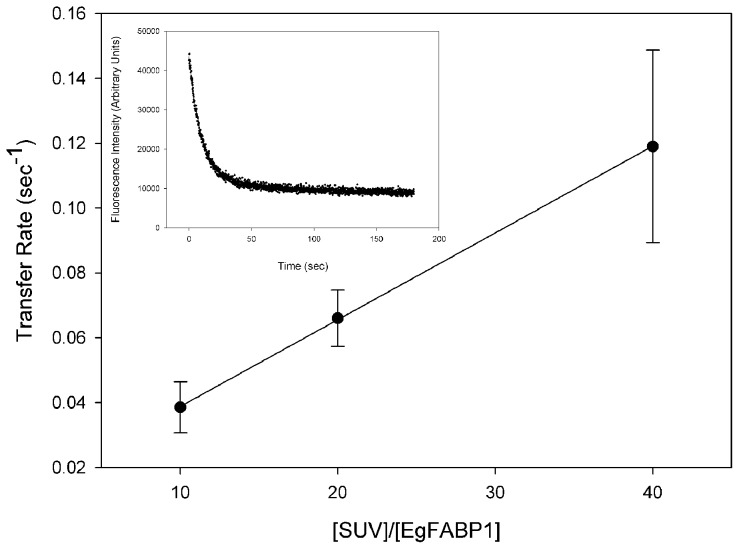
Effect of acceptor membrane concentration on 12AS transfer from EgFABP1 to EPC/NBD-PC SUVs. SUVs were added ranging from 1∶10 mol∶mol to 1∶40 mol∶mol EgFABP1∶SUVs to 15∶1 mol∶mol EgFABP1∶12AS complex. Fatty acid transfer rates were directly monitored by the time-dependent decrease in anthroyloxy group fluorescence, as described in Materials and Methods. Average of four different experiments ± SD are shown. The inset shows an example of the kinetic trace obtained when combining EgFABP1-12AS with membranes that contain NBD-PC.

In a collisional transfer, the limiting step is the effective protein-membrane interaction, and the rate increases as the acceptor membrane concentration increases. In a diffusional mechanism in which the rate limiting step is the dissociation of the protein-ligand complex, no change in rate is observed [33], [37], [38], [44], [45], [47]. The values of K_d_ and K_P_ were used to set the conditions for the transfer assay. The proportion of protein and ligand was such that less than 1% of AOFA remained free in the preincubation solution. On the other hand, K_P_ value was used to calculate the final concentrations of protein and SUVs for which unidirectional transfer prevailed. Figure 4 shows that when constant concentrations of the EgFABP1-12AS donor complexes were mixed with increasing concentrations of EPC-SUV, the 12AS transfer rate from EgFABP1 to EPC-SUV increased proportionally to vesicle concentration in the SUV: EgFABP1 ratios (10∶1 to 40∶1) tested. In these conditions, the increase in transfer rate ranged from 0.04±0.01 sec^−1^ to 0.12±0.03 sec^−1^. These results strongly suggest that the FA transfer from EgFABP1 occurs via a protein-membrane interaction rather than by simple aqueous diffusion of the free ligand.

### Effect of phospholipids charge on AOFA transfer from EgFABP1 to membranes

**Figure 5:**
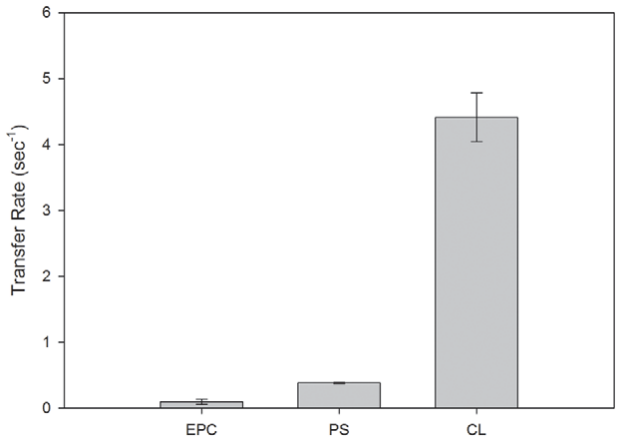
Effect of acceptor membrane surface charge on 12AS transfer rates from EgFABP1 to SUVs. Transfer rate from EgFABP1∶12AS (15∶1 mol∶mol) complex to 1∶20 mol∶mol of EPC/NBD-PC SUVs containing no other phospholipids (zwitterionic vesicles), 25 mol % brain PS (single negative charge) or CL (double negative charge) were measured. Averages from two different experiments ± SD are shown.

**Figure 6:**
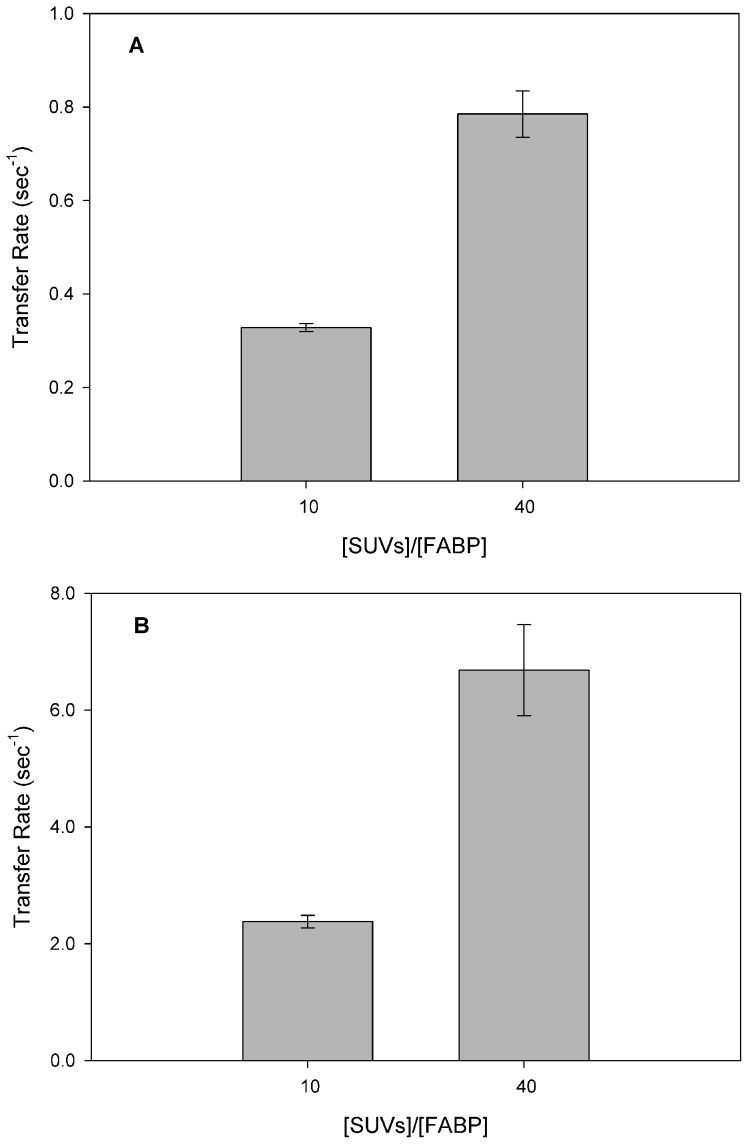
Effect of acceptor membrane concentration on 12AS transfer rates from EgFABP1 to charged SUVs. Transfer rate from EgFABP1∶12AS (15∶1 mol∶mol) complex to 1∶10 mol∶mol or 1∶40 mol∶mol EgFABP1∶EPC/NBD-PC SUVs containing either (A) 25 mol % PS or (B) CL were measured. Averages from two different experiments ± SD are shown.

Considering the hypothesis that FA transfer from EgFABP1 occurs by collisional contact with an acceptor membrane, changes in membrane properties should modify the transfer rate. If the mechanism relied on aqueous diffusion alone, then the characteristics of acceptor membranes should be irrelevant to the transfer rate, since the rate-determining step in such a transfer process (ligand dissociation into the aqueous phase) is a physically and temporally distinct event from processes involving the membrane. Figure 5 shows that 12AS transfer rate from EgFABP1 to membranes increased when acceptor membranes contained 25% of negatively charged phospholipids (PS or CL). In agreement with the behaviour we have previously observed for collisional mammalian FABPs [38], [44], [45], [47], EgFABP1 shows a large increase in FA transfer rate to CL vesicles compared with zwitterionic vesicles. To investigate further the effect of negative charge of the acceptor vesicles on the FA transfer mechanism from the protein, we analysed the modification of transfer rates with increasing concentrations of negatively charged acceptor vesicles. The rate of FA transfer from EgFABP1 always, and independently of the net charge of the vesicles, showed the classical proportional increase in transfer rate with acceptor concentration (Figure 6).

### Effect of ionic strength on AOFA transfer from EgFABP1 to membranes

**Figure 7:**
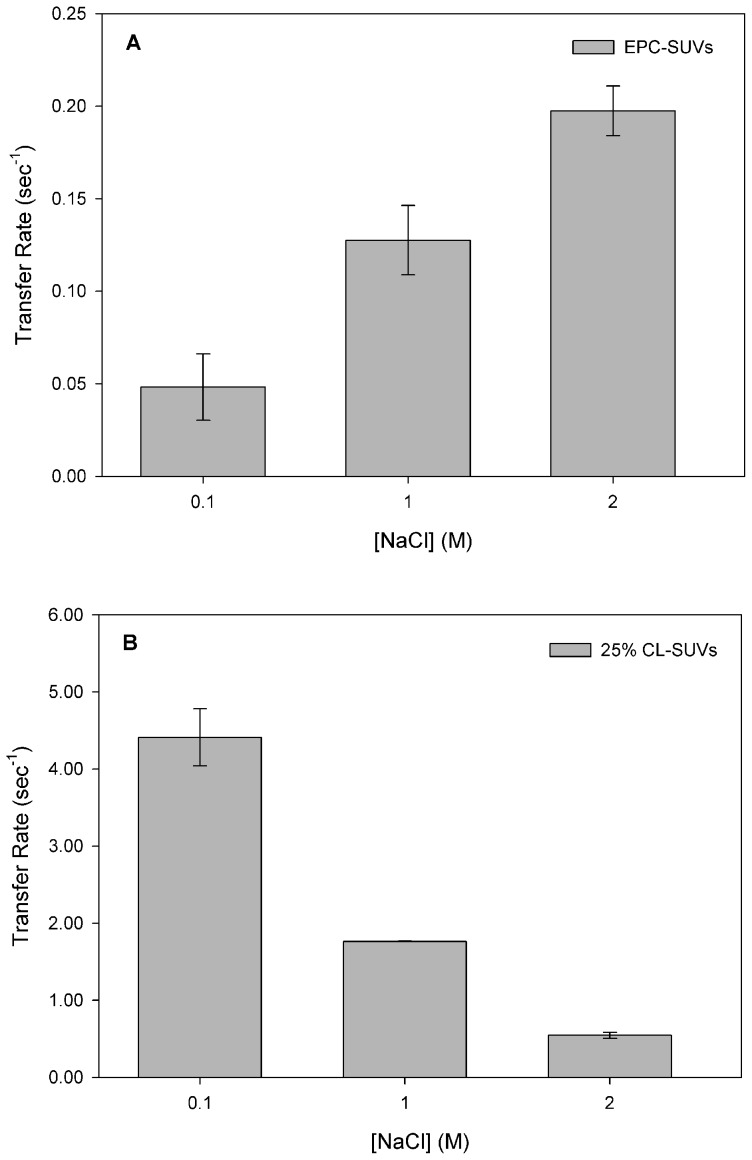
Effect of ionic strength of buffer on 12AS transfer rate from EgFABP1 to SUVs. Transfer rates from EgFABP1∶12AS (15∶1 mol∶mol) complex to 1∶20 mol∶mol of EgFABP1∶EPC/NBD-PC SUVs (A) or EPC/NBD-PC SUVs containing 25 mol % CL (B) in presence of increasing concentrations of NaCl were measured. Average of four different experiments ± SD are shown (except data corresponding to 2M NaCl, which derive from two independent experiments).

Transfer of 12AS from EgFABP1 to membranes was examined as a function of increasing concentrations of NaCl. The results show that an important increase in transfer rate from EgFABP1 to neutral membranes was detected with increasing ionic strength of the aqueous phase (Figure 7A). It is generally thought that electrostatic interactions at surfaces are diminished and hydrophobic interactions are stimulated as a function of increasing ionic strength. The effect of ionic strength on the rate of AOFA transfer from EgFABP1 to zwitterionic vesicles suggests that the elimination of electrostatic interactions by salt shielding is compensated by an increase in hydrophobic interactions.

When negative charge was added to the acceptor lipid vesicles, a drastic decrease was observed at high salt concentrations (Figure 7B). As shown in Figure 5, EgFABP1 exhibited approximately a 60-fold increase in AOFA transfer rate to CL vesicles compared with EPC vesicles at low ionic strength. Upon increasing the ionic strength, a marked decrease from the very high values observed at low ionic strength was found (Figure 7B). This suggests a masking of electrostatic interactions, which play a very important role at low ionic strength, caused by the high salt content of the buffer.

### Direct competitive interaction between EgFABP1 and cytochrome c with membranes

**Figure 8:**
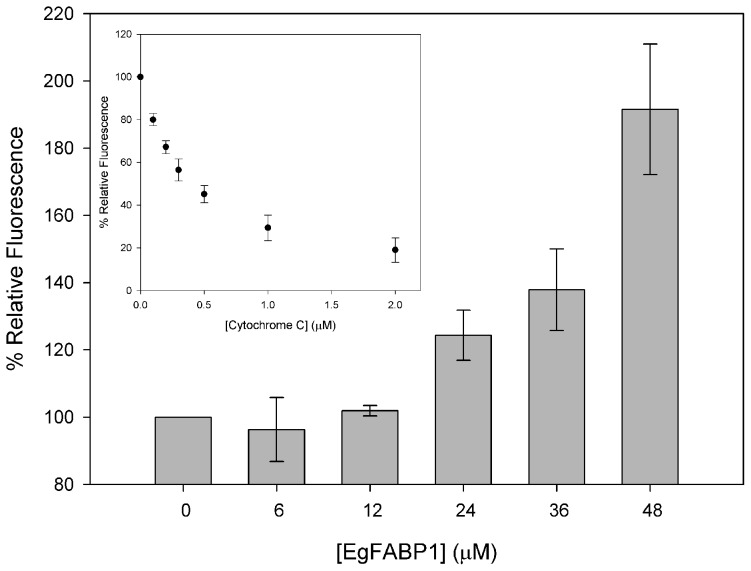
Inhibition of cytochrome c binding to anionic membrane by EgFABP1. Addition of increasing amounts of cytochrome c to 15 µM SUVs containing 64% EPC, 10% EPE, 25% CL and 1% DPE caused a decrease in dansyl fluorescence emission. DPE-containing vesicles were >70% quenched upon addition of 1 µM cytochrome c in absence of EgFABP1 (inset). For the inhibition of binding analysis, 15 µM SUVs were incubated with increasing concentrations of EgFABP1 (0–48 µM), 1 µM cytochrome c was subsequently added, and the relative increase in dansyl fluorescence emission at 520 nm was monitored. Results are expressed as the percent relative fluorescence intensity, where 100% represents the relative fluorescence intensity of SUVs incubated in the presence of cytochrome c, but without EgFABP1. Results are the average of two experiments ± SD.

FA transfer experiments suggest that the interaction of EgFABP1 with membranes is sensitive to surface charge density. As cytochrome c is known to interact as a peripherally associating protein with acidic membranes [48], we analysed the ability of EgFABP1 to compete with cytochrome c for binding to membranes containing CL. Cytochrome c quenches dansyl fluorescence in a concentration-dependent manner (ref. [49] and Figure 8). Results show that preincubation of CL-containing vesicles with EgFABP1 was effective in preventing cytochrome c binding in a concentration-dependent manner (Figure 8). When EgFABP1 (48 µM) was added to CL-containing SUVs, the dansyl fluorescence was twice that obtained in the absence of EgFABP1 and with 1 µM cytochrome c.

## Discussion

We show that recombinant EgFABP1 is able to bind FAs of different chain lengths from *E. coli*, mainly palmitic and stearic acids. This is clearly an incomplete inventory of ligands that it may transport in the parasite, but it does illustrate the propensity of the protein to bind FAs when exposed to an environment rich in a wide range of small hydrophobic compounds. The analysis of the natural ligands bound by EgFABP1 in the parasite environment is currently being undertaken in our laboratory.

Our main finding in this work was that the protein engages in a collisional mechanism in ligand transfer, as do various FABP isoforms from mammals, and one from Schistosomes, that have been investigated in this way [8], [11], [12]. This involvement of direct contact between protein and membrane for this transfer was found by altering electrostatic and hydrophobic conditions in the transfer experiments. The results indicated that the interaction event is mediated by both charge and hydrophobic factors, and it would seem reasonable that the initial interaction is ionic, between the protein and charged phospholipid headgroups, followed by direct, transient hydrophobic interaction with the apolar layer of the membrane. The interaction of the protein with membranes has also been demonstrated by the competition with cytochrome c for membrane binding.

The tertiary structure of EgFABP1 is virtually superimposable on FABPs that engage in collisional transfers [27], in which the two alpha-helices adjacent to the portal of ligand entry in FABPs are important in engaging contact with membranes [38]. It may be no coincidence that EgFABP1 has, like these other collisional FABPs, a prominent pair of bulky hydrophobic amino acid sidechains (Phe27, Val28) extending into solvent from helix II, immediately adjacent to the portal. Such a ‘sticky finger’ could attract and orient ligand for entry into the protein, or be involved in the protein's interaction with membranes or other proteins [50].

Our results suggest that EgFABP1 is likely to be an active participant in the transport and exchange of lipids *in vivo*, which could involve uptake of FAs directly from, and delivery to, membranes within the parasite, potentially resourcing the developing protoscoleces within the hydatid cysts. This might also be the case for Antigen B, which belongs to a new family of hydrophobic ligand binding proteins of cestodes and has been proposed as a lipoprotein involved in lipid trafficking [46], [51]. Furthermore, our proteolysis experiments with EgFABP1 and the analysis of CD spectra of apo- and holo-forms indicated that ligand binding would induce a conformational change in the protein. Such a change might modify the mechanism of interaction of EgFABP1 with membranes to facilitate upload or download of their cargo. A conformational change could also function as a signal to target the protein to different destinations, as has been suggested for other members of the FABP family [52], [53]. The possibility that it also interacts with host cell membranes is more contentious, particularly since EgFABP1 does not have a secretory leader peptide, as is also the case for FABPs from any group of animals other than nematodes [5], [6], so should be confined to the interior of cells. However, if EgFABP1 appears in cyst fluid *in vivo* and in excretion/secretion products of protoscoleces [15], [16] (but not as a result of cell damage during fluid collection or imperfect culture conditions in the collection of excretion/secretion products) then the possibility that it does interact with host cells beyond the cyst wall must be considered. Host proteins are known to cross hydatid cyst walls [15], so it is conceivable that this permeability (if a unidirectional transfer system is not in operation) could mean that EgFABP1 leaves the cyst to interact with host membranes for return to the parasite, or to deliver lipids to host tissues for immunomodulation. These hypotheses remain to be tested. In this regard, future studies should also include protein interaction analysis with membranes that mimic parasite and host composition.

This work is a first approach to understand the functional properties of EgFABP1 and constitutes the basis for further expanding our knowledge about this protein. This has been the case for other members of the FABP family, where this kind of studies has contributed to the understanding of the mechanisms of ligand transfer to membranes, protein-membrane and protein-protein interactions [8], [54].

## Supporting Information

Figure S1 **Circular dichroism spectra of apo- and holo-EgFABP1.** (A) CD spectra in the FAR UV region of apo-EgFABP1, palmitic acid-EgFABP1 and oleic acid-EgFABP1. Results show that ligand binding does not induce significant changes in the secondary structure of the protein. (B) CD spectra in the near UV region of the same samples. These results show that the spectrum of EgFABP1 changes upon ligand binding, especially when oleic acid is bound to the protein, indicating that the environment of the aromatic aminoacids is modified.
(TIF)

Protocol S1 **Circular dichroism spectra acquisition.** Circular dichroism spectra in the near (250–320 nm) and far (200–250 nm) UV spectra of EgFABP1 in its apo-form, and bound to either palmitic or oleic acid.
(DOCX)
