# Supplementary figures and images for: Direct Interaction between EgFABP1, a Fatty Acid Binding Protein from Echinococcus granulosus, and Phospholipid Membranes
Source: PLoS Negl Trop Dis. 2012 Nov 15;6(11):e1893. doi: 10.1371/journal.pntd.0001893 (PMC3499409; doi:10.1371/journal.pntd.0001893)

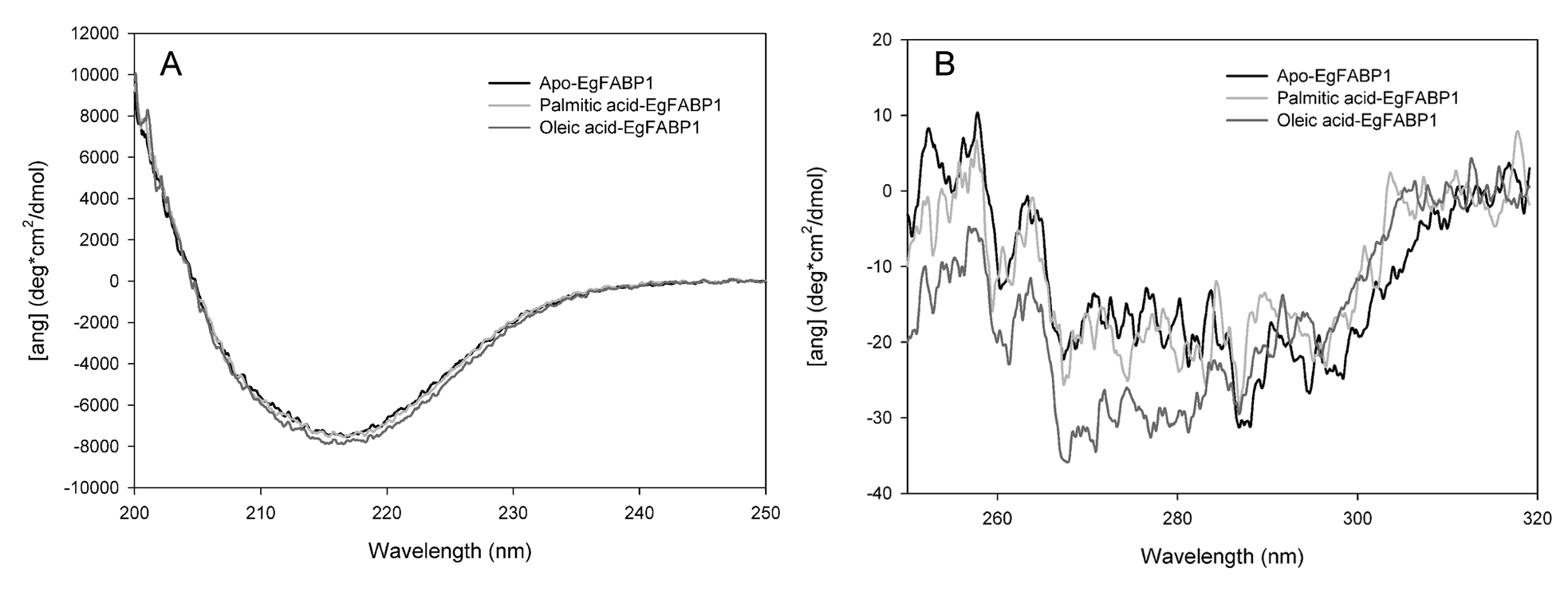

Supplement: Figure S1 — Circular dichroism spectra of apo- and holo-EgFABP1. (A) CD spectra in the FAR UV region of apo-EgFABP1, palmitic acid-EgFABP1 and oleic acid-EgFABP1. Results show that ligand binding does not induce significant changes in the secondary structure of the protein. (B) CD spectra in the near UV region of the same samples. These results show that the spectrum of EgFABP1 changes upon ligand binding, especially when oleic acid is bound to the protein, indicating that the environment of the aromatic aminoacids is modified. (TIF) [file pntd.0001893.s001.tif]
