# Supplementary material for: Direct Interaction between EgFABP1, a Fatty Acid Binding Protein from Echinococcus granulosus, and Phospholipid Membranes
Source: PLoS Negl Trop Dis. 2012 Nov 15;6(11):e1893. doi: 10.1371/journal.pntd.0001893 (PMC3499409; doi:10.1371/journal.pntd.0001893)
Supplement: Protocol S1 — Circular dichroism spectra acquisition. Circular dichroism spectra in the near (250–320 nm) and far (200–250 nm) UV spectra of EgFABP1 in its apo-form, and bound to either palmitic or oleic acid. (DOCX) [file pntd.0001893.s002.docx]

Protocol S1

**Circular Dichroism spectra**

CD spectra were recorded on a Jasco J-810 spectropolarimeter. Data in the near-UV (250-320 nm) or in the far-UV (200-250 nm) regions were collected using a 10 mm or a 1 mm path cuvette, respectively. A scan speed of 20 nm/min with a time constant of 1 s was used. EgFABP1 (31.6 µM) was dissolved in PBS buffer. The holo-forms of EgFABP1 were obtained incubating the protein with either palmitic or oleic acid at a [ligand] to [protein] ratio of 4. Each spectrum was measured at least three times, and the data were averaged to reduce noise. Molar ellipticity was calculated as described elsewhere [1].

[1] Schmidt F (1989) Spectral methods of characterizing protein conformation and conformational changes. In: Creighton TE, editors. Protein Structure: A Practical Approach. IRL Press. p251
